# Supplementary material for: MiR-302 Regulates Glycolysis to Control Cell-Cycle during Neural Tube Closure
Source: Int J Mol Sci. 2020 Oct 13;21(20):7534. doi: 10.3390/ijms21207534 (PMC7589003; doi:10.3390/ijms21207534)
Supplement: Supplementary file 1 [file ijms-21-07534-s001.pdf]

Supplementary Figures

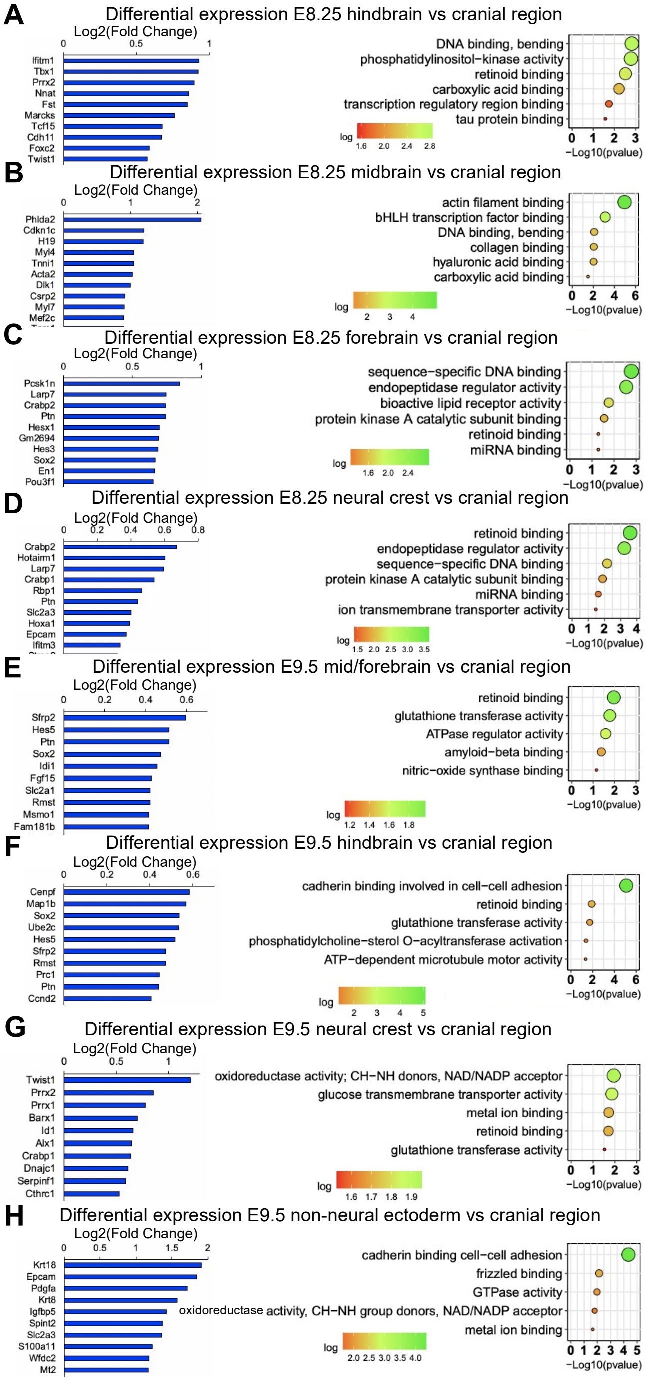

Supplemental Figure 1. Metabolism and differentiation are coupled during neurulation. (**A**) Bar plot of differentially expressed genes for the comparison of each ectoderm-derived cell population against the rest of the ectoderm-derived and non-ectoderm-derived cells of the cranial region, followed by molecular gene ontology for the E8.25 hindbrain, (**B**) E8.25 midbrain, (**C**) E8.25 forebrain, (**D**) E8.25 neural crest, (**E**) E9.5 forebrain/midbrain, (**F**) E9.5 hindbrain, (**G**) E9.5 neural crest, and (**H**) E9.5 non-neural ectoderm.

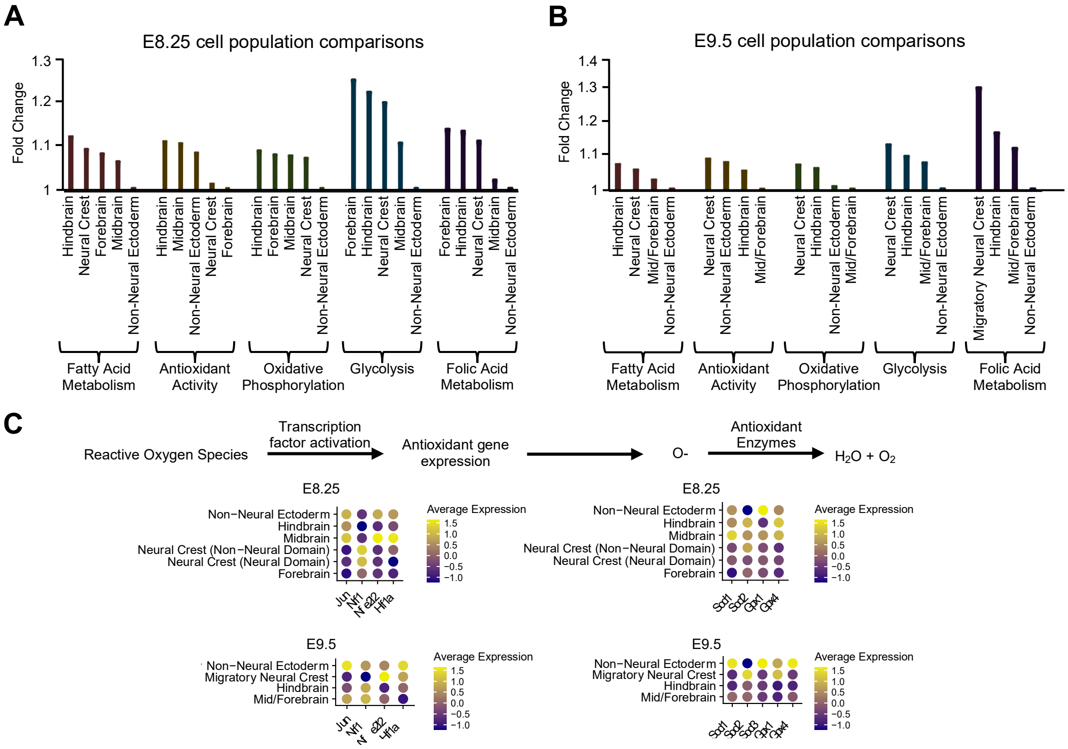

Supplemental Figure 2. Single-cell sequencing reveals metabolic contributions to neural tube closure. (**A**) Bar plot showing gene enrichment for metabolic pathways at E8.25 and (**B**) E9.5. Average expression of each cell population was normalized to that with the lowest expression for each metabolic pathway. (**C**) Dot plots showing expression of redox transcription factors and antioxidant enzymes in ectoderm-derived cell populations at E8.25 and E9.5.

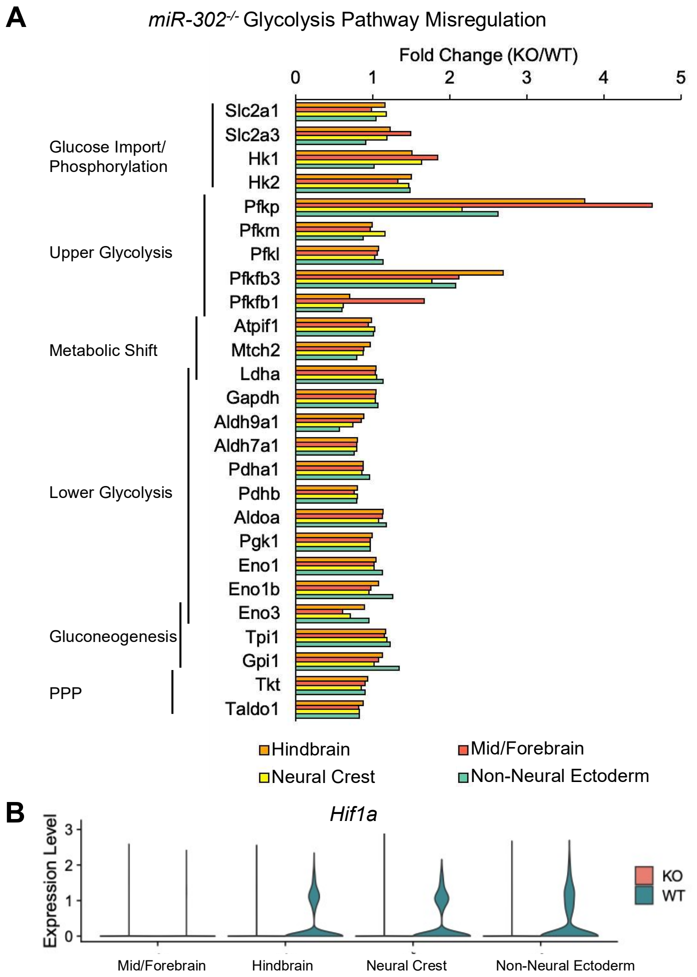

Supplemental Figure 3. Upper glycolysis is regulated by *miR-302*. (**A**) Bar plot of the misregulated genes of the various glycolysis pathway divisions in the *miR-302* knockout ectoderm-derived cell populations. (**B**) Violin plot of Hif1a misregulation in the *miR-302* knockout.

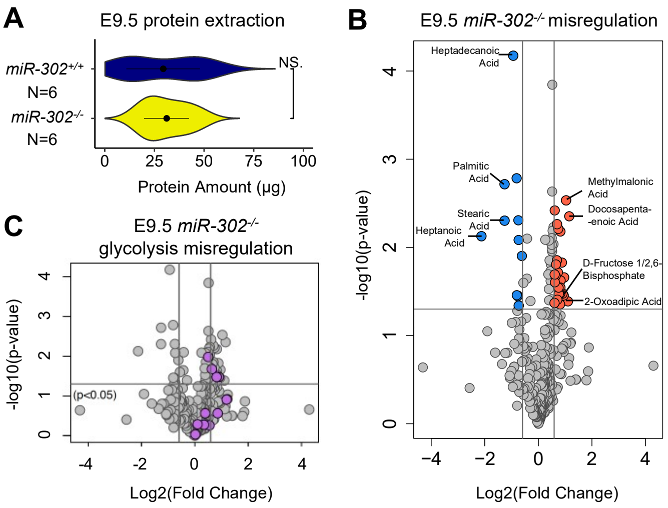

Supplemental Figure 4. Loss of *miR-302* leads to an increase of glycolytic intermediates. (**A**) Violin plot of the total amount of protein harvested from the cranial regions of wildtype and *miR-302* knockout embryos for mass spectrometry. (**B)** Volcano plot showing significantly up- and down-regulated metabolites of the *miR-302* knockout. (**C**) Volcano plot showing the upregulation of glycolysis metabolites.

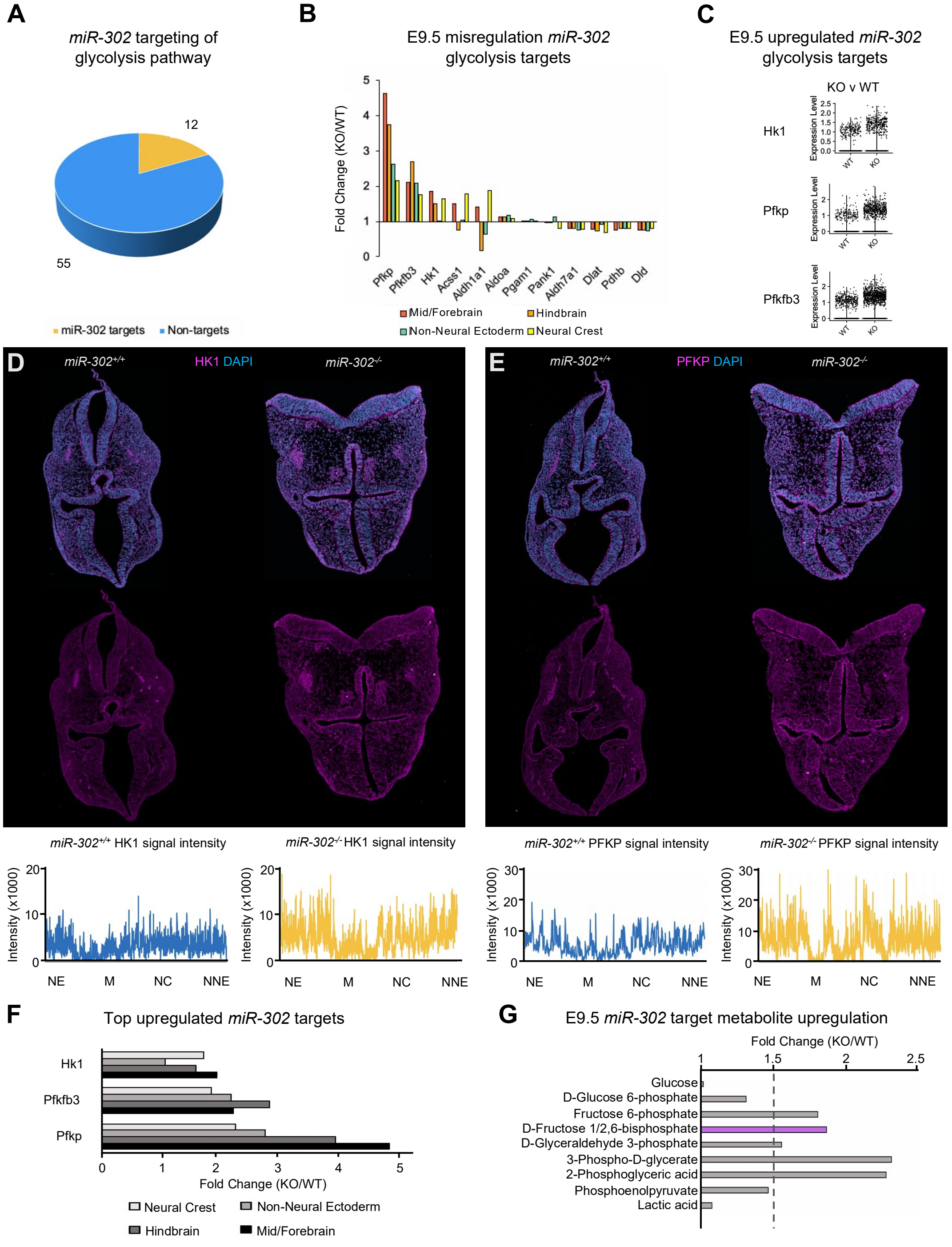

Supplemental Figure 5. *miR-302* targets *Pfkp*, *Pfkfb3*, and *Hk1* to regulate upper glycolysis. (**A**) Pie chart showing that *miR-302* targets 12 genes within the glycolysis pathway. (**B**) Bar plot showing the upregulation of *miR-302* targets within the glycolysis pathway across each ectoderm-derived cell population. (**C**) Dot plots showing expression of Pfkp, Pfkfb3, and Hk1 in wildtype and *miR-302* knockout embryos. (**D**) Immunofluorescence for HK1 and (**E**) PFKP in wildtype and *miR-302* knockout embryos. Intensity measurements were taken across comparable regions of embryos and was plotted to highlight increase in intensity of signal upon *miR-302* deletion. (**F**) Bar plot of the top three upregulated *miR-302* targets Pfkp, Pfkfb3, and Hk1 in each ectoderm-derived cell population. (**G**) bar plot showing the only significantly upregulated glycolysis metabolite with a fold change >1.5 is that produced by upregulated *miR-302* target Pfkfb3.

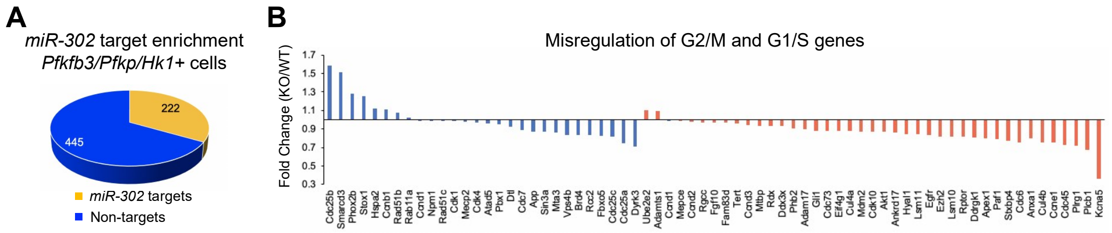

Supplemental Figure 6. *miR-302* targets *Pfkp*, *Pfkfb3*, and *Hk1* to regulate cell proliferation. (**A**) Pie chart of the number of *miR-302* targets and non-targets obtained as differentially expressed genes from the differential expression analysis of *Pfkfb3*/*Pfkp*/*Hk1*+ cells. (**B**) Bar plot showing the misregulation of cell cycle transition genes upon *miR-302* deletion.

Supplementary Tables

Supplementary Table 1: Genes used for identification of ectoderm-derived cell populations.

Neural Crest	Fore/Midbrain	Hindbrain	Non-neural Ectoderm
Sox9	Otx2	Pax3	Cdh1
Pax3	Cdh2	Sox1	Krt8
Pax7	Pax6	Cdh2	Krt18
Sox10	Pax5	En1	Epcam
Tfap2a	Fgf15	Fgf15	Trp63
Cdh1		En2	Grhl2
Ets1			
Snai2			
Msx1/2			
Id3			
